# Multimodal Dementia Prediction With Large Language Models: Cross-Attention Over Text, Audio, and Image

**DOI:** 10.2196/93279

**Published:** 2026-09-21

**Authors:** Felix Agbavor, Hualou Liang

**Affiliations:** 1 School of Biomedical Engineering and Science, Drexel University, Philadelphia, PA, United States; 2 Division of Artificial Intelligence and the Humanities, The Hong Kong Polytechnic University, HHB717, 7/F, 8 Hung Lok Road, Hung Hom, Kowloon, China (Hong Kong), 852 2766-7697; 3 Department of Language Science and Technology, The Hong Kong Polytechnic University, Kowloon, China (Hong Kong); 4 Departments of Data Science and Artificial Intelligence, The Hong Kong Polytechnic University, Kowloon, China (Hong Kong)

**Keywords:** Alzheimer disease, multimodal fusion, speech analysis, cognitive assessment, cross-attention

## Abstract

**Background:**

Alzheimer disease (AD) is a leading cause of dementia, and there is growing interest in scalable approaches for early screening using speech-based tasks. While prior work has demonstrated promising results using either transcript-based language features or acoustic cues, most approaches remain unimodal or rely on simple fusion strategies that do not explicitly consider interactions across modalities.

**Objective:**

In this study, we propose an attention-based trimodal fusion framework that integrates text, audio, and image representations of the Cookie Theft picture, which serves as the shared visual stimulus in the picture-description task.

**Methods:**

Our method uses a new bidirectional cross-attention mechanism to achieve a unified multimodal embedding for downstream tasks. We evaluate the approach on 2 tasks: AD detection by classifying whether the participant has AD or not, and AD severity assessment by predicting Mini-Mental Status Examination cognitive scores.

**Results:**

On the AD detection task, trimodal fusion achieves the best overall performance (*F*_1_-score=0.8667, area under the receiver operating characteristic curve=0.9032), outperforming unimodal baselines, bimodal fusion, and conventional early or late fusion methods. For AD severity assessment, the proposed multimodal representation reduces prediction error of root mean squared error to about 4.20, improving over both unimodal and bimodal fusion settings. We further perform the ablation analysis to show that bidirectional cross-attention consistently outperforms conventional unidirectional cross-attention.

**Conclusions:**

These results demonstrate that attention-based multimodal fusion can enhance dementia prediction from picture-description responses and provide a strong foundation for developing multimodal cognitive screening pipelines.

## Introduction

Alzheimer disease (AD) is a progressive neurodegenerative disorder and a leading cause of dementia worldwide, with a growing public health impact due to aging populations [1,2]. Early screening and continuous monitoring are critical for timely intervention, planning, and patient support [3]. However, conventional clinical assessments remain resource-intensive, often requiring specialized expertise, controlled administration environments, expensive clinical facilities, and repeated in-person visits [4,5]. These limitations motivate scalable, accessible, noninvasive, and low-burden approaches for cognitive screening that can be deployed in realistic settings.

Spontaneous speech has emerged as a particularly promising biomarker for dementia because it reflects a wide range of cognitive-linguistic processes, including lexical retrieval, syntactic organization, semantic coherence, and executive control [6-8]. A substantial body of work demonstrates that spontaneous speech contains clinically meaningful signals for AD screening and diagnosis. However, most existing studies remain unimodal, relying solely on either textual features derived from transcripts or acoustic features extracted from audio, thereby neglecting complementary information that is critical for robust inference. More recent approaches have explored bimodal models (eg, audio + text), which generally outperform unimodal baselines by aligning linguistic and acoustic cues [8-10]. Despite these advances, bimodal analyses primarily focus on local speech characteristics such as pauses and lexical diversity and often fail to capture global discourse-level phenomena, including disrupted topic maintenance and cross-modal inconsistencies between spoken narratives and visual image.

To address these limitations, we introduce image information into AD prediction by jointly modeling text, audio, and image representations in the Cookie Theft picture-description task. Because the Cookie Theft picture is identical across all participants, the visual modality is not intended to encode participant-specific variability; instead, it provides shared visual context that grounds the spoken narrative during multimodal fusion. This visual context helps relate the narrative to the scene being described and serves as a semantic anchor for fusion. Each modality contributes different information. Text captures lexical choice, semantic content, coherence, and informativeness of the spoken description. Audio contributes complementary paralinguistic cues, including pauses, hesitation, rhythm, fluency, and other prosodic features not fully preserved in transcripts. The Cookie Theft picture provides shared visual context that grounds the narrative and helps assess how well the spoken description aligns with the scene.

Multimodal integration is commonly approached through 3 fusion strategies: early (data-level), intermediate (joint), and late (decision-level) fusion. Early fusion projects features from different modalities into a single shared space, but it often fails to capture higher-order cross-modal interactions. Late fusion, by contrast, aggregates the outputs of modality-specific models, preserving individual modality strengths while overlooking deeper interdependencies among them [11-13]. Intermediate, or joint, fusion offers a principled compromise by explicitly modeling interactions across modalities during representation learning. In this context, Transformer-based cross-attention has emerged as a key mechanism for capturing fine-grained and long-range intermodal relationships [14]. Intuitively, cross-attention allows one modality to look at another and selectively use the parts that are most relevant for the task. For example, a text representation can attend to complementary information in the audio or visual representation, so the fused embedding reflects not only what was said, but also how it was spoken and how well it aligns with the Cookie Theft picture context.

Cross-attention has been widely adopted in language-vision representation learning [8,15], audio-text integration [11], and more recently in dementia prediction [16]. However, existing approaches typically rely on unidirectional cross-attention, in which one modality queries another, resulting in asymmetric information flow. To overcome this limitation, we propose a bidirectional cross-attention mechanism that enables symmetric and iterative information exchange across modalities. In this design, each modality both attends to and is updated by the other, allowing representations to be refined in both directions. This iterative bidirectional interaction facilitates richer cross-modal integration [8,15], promotes balanced modality contributions, and mitigates the risk of modality dominance. Building on this perspective, our trimodal fusion architecture leverages bidirectional cross-attention to produce unified embeddings that capture both complementary evidence and cross-modal agreement, rather than relying on fixed or heuristic fusion rules. Simply put, rather than allowing only one modality to query information from another, bidirectional cross-attention enables both modalities to interact and refine each other, resulting in a fused representation derived from 2-way information exchange rather than 1-way conditioning.

**Figure 1.**
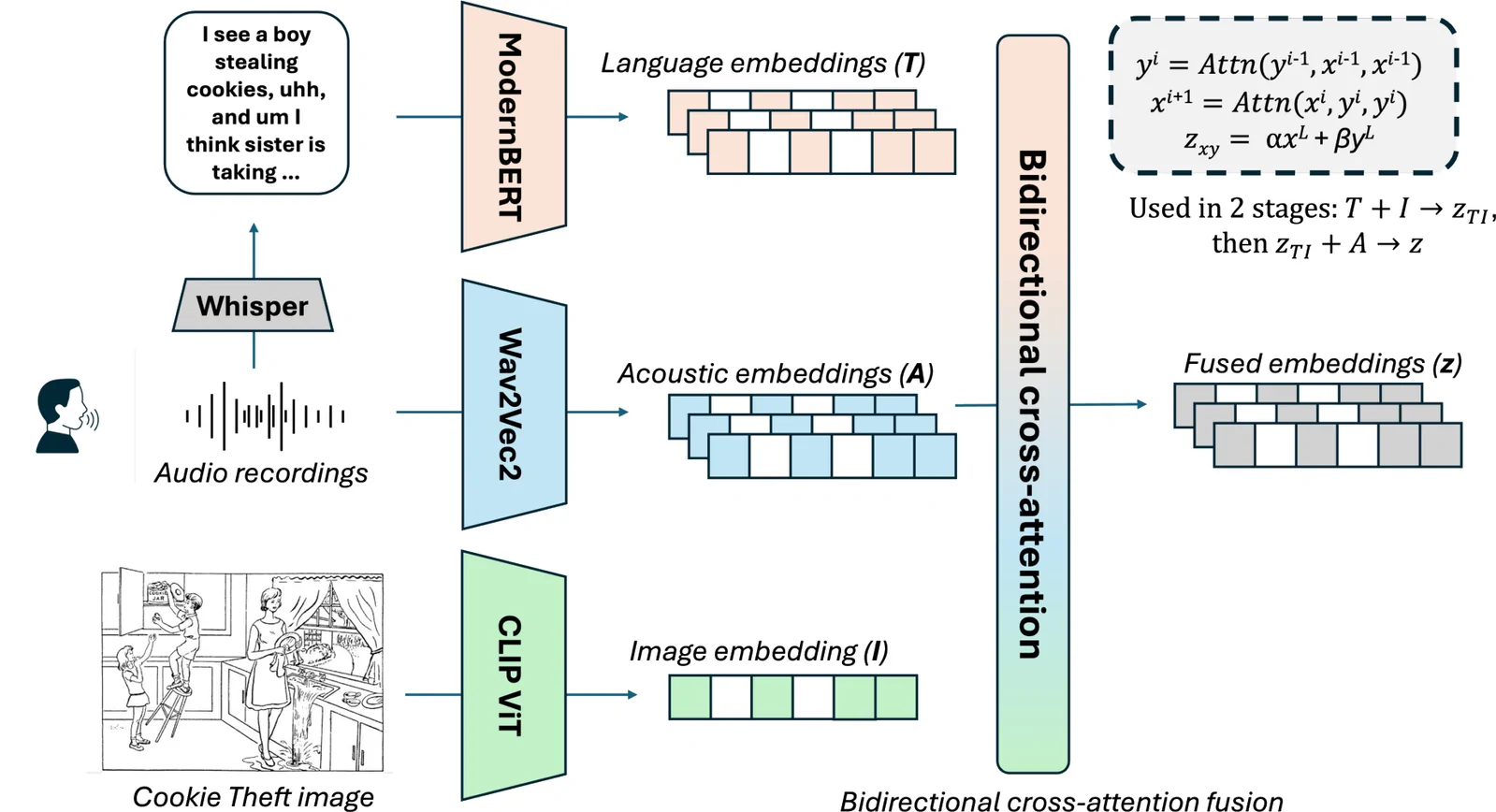
Proposed trimodal fusion architecture for dementia prediction. Pretrained 768-dimensional embeddings are extracted from text (ModernBERT), audio (Wav2Vec 2.0), and the Cookie Theft picture (contrastive language–image pretraining [CLIP] ViT-L/14). Fusion is performed in 2 stages for clarity. In stage 1, text and image are fused through a bidirectional bimodal cross-attention block to obtain an intermediate representation, *TI*. In stage 2, this intermediate *TI* representation is fused with audio through the same bidirectional fusion block to produce the final multimodal embedding, *z*, which is used for downstream classification and regression. The inset illustrates the generic bimodal fusion module used in each stage, where 2 modality embeddings are updated sequentially across layers by attending to one another, enabling reciprocal information exchange beyond standard 1-way cross-attention.

Taken together, we propose a trimodal embedding-level fusion architecture that integrates text, audio, and image through bidirectional cross-attention (Figure 1). The resulting fused embedding is used for downstream AD detection and cognitive score prediction. We evaluate this approach on the ADReSSo 2021 picture-description task against unimodal baselines, bimodal fusion models, and conventional early- and late-fusion strategies.

Our main contributions are as follows:

- We introduce a staged bidirectional cross-attention fusion framework for integrating text, audio, and image representations in the AD picture-description setting.
- On the ADReSSo 2021 picture-description task, we show that trimodal fusion improves over unimodal baselines and outperforms bimodal fusion for both AD detection and Mini-Mental Status Examination (MMSE) prediction.
- We show that the proposed fusion framework performs better than conventional fusion strategies, including early fusion and late fusion.
- We further show that bidirectional cross-attention yields more effective and stable performance than a standard 1-way cross-attention under the same task setting.

## Methods

### Dataset Description

The dataset used in this study is derived from the ADReSSo 2021 Challenge and consists of speech recordings from a picture-description task [8], where cognitively normal participants and individuals diagnosed with AD were asked to describe the Cookie Theft image from the Boston Diagnostic Aphasia Examination [17,18]. In total, the dataset contains 237 recordings, split into a 70/30 train–test partition with demographic balancing, resulting in 166 samples for training and 71 for testing. Within the training set, there are 87 AD and 79 non-AD (healthy control) recordings.

A key strength of ADReSSo is its focus on reducing common sources of bias in dementia screening benchmarks. The dataset was carefully curated to mitigate confounds such as repeated speech samples from the same individual, variation in recording quality, and imbalances in age and gender. Demographic matching was performed using a propensity-score procedure as described by Luz et al [8], yielding standardized mean differences below 0.001 for both age and gender, which helps ensure that downstream model performance reflects disease-related signal rather than demographic artifacts.

### Dataset Preprocessing and Feature Representation

We preprocess the ADReSSo picture-description recordings to obtain aligned audio, text, and image representations, which serve as inputs to our multimodal fusion architecture. For the audio modality, we extract embeddings using Wav2Vec 2.0 [19], a self-supervised speech representation model trained to learn rich acoustic features directly from raw waveforms. Given an input recording, we obtain a sequence of frame-level hidden states and apply mean pooling over time to produce a fixed-dimensional 768-D audio embedding per sample.

For the text modality, we first transcribe each recording using OpenAI Whisper [20], which provides robust automatic speech recognition for spontaneous speech. To better preserve clinically relevant phenomena commonly observed in AD speech, we supply an initial decoding prompt (*“*Umm, Uhh, let me think like, hmm... Okay, here’s what I’m, like, thinking*."*) that encourages retention of fillers, disfluencies, repetitions, and incomplete phrases, rather than normalizing them away. We then encode the resulting transcripts using ModernBERT [21], a modern bidirectional Transformer encoder optimized for producing strong sentence-level representations. Similar to the audio pipeline, we derive a fixed-length 768-D text embedding for each transcript, providing a compact semantic representation that captures content, coherence, and lexical organization.

For the image modality, we represent the Cookie Theft stimulus using contrastive language–image pretraining (CLIP; ViT-L/14), a vision-language model trained via contrastive learning to align visual and textual representations in a shared embedding space. We extract the image encoder output and use a 768-D image embedding, which provides a high-level description of the visual scene structure and salient objects.

### Attention-Based Multimodal Fusion

A central component of our fusion approach is bidirectional cross-attention, where each modality is updated by attending to the others, allowing text, audio, and image embeddings to exchange information in both directions rather than being combined through simple concatenation. Unlike simple fusion methods (eg, concatenation), cross-attention allows the model to learn which parts of one representation are most relevant given the other representation, which is especially important in dementia screening where different modalities may carry complementary cues. Formally, we use the standard attention operation represented by $Attention\left(Q,K,V\right)\text{=}softmax\left(\frac{QK^{T}}{\sqrt{d_{k}}}\right)V$, where *Q* (queries) represents what we want to “look for,” while *K* (keys) and *V* (values) represent the information we want to retrieve from.

Intuitively, bidirectional cross-attention can be understood as a 2-way exchange between modalities. In one direction, the text representation asks which parts of the audio or image are most relevant for interpreting what was said; in the other direction, the audio or image representation is also updated by attending back to the text. This allows the fused representation to reflect not only the content of the spoken description, but also how it was delivered and how well it aligns with the shared Cookie Theft picture context. In practice, the bidirectional design helps the model avoid treating one modality as fixed context for another and instead encourages reciprocal refinement of the modality embeddings across layers.

To combine information from text, image, and audio in a way that goes beyond simple concatenation, we propose an attention-based fusion module (Figure 1) that learns how modalities complement each other when predicting AD status and cognitive outcomes. The key idea is that each modality carries different but related information: text reflects linguistic content and coherence, audio captures acoustic patterns such as hesitation and fluency, and the image provides the visual context that grounds the picture-description narrative. Rather than assuming each modality contributes equally, our fusion approach learns to emphasize the most informative signals dynamically.

Our architecture performs multimodal fusion using bidirectional cross-attention, enabling each modality to iteratively incorporate information from the others. Rather than simply concatenating embeddings, the model allows the text representation to attend to complementary cues from the image and audio, while the non-text modalities also attend back to the evolving text representation. This bidirectional exchange supports richer alignment between what is said, how it is spoken, and the visual context of the picture-description stimulus. As a result, the fusion module produces a single multimodal embedding that reflects both modality-specific evidence and cross-modal dependencies, which is then used for downstream AD classification and its severity assessment.

For the implementation, we use a multilayer attention block that alternates between the 2 inputs, allowing one modality to “look at” and incorporate information from the other. Intuitively, this helps the model learn cross-modal interactions. For example, how acoustic hesitation patterns relate to lexical choice or how a visually grounded narrative differs between cognitively normal and impaired speakers. The final output is a single fused embedding per sample, which is passed to downstream classifiers or regressors for AD detection and its severity assessment.

### Training Regimen

We train the proposed trimodal fusion model using the ADReSSo training split and further reserve 20% of the training data as a validation set for model selection and early stopping. This validation split is created randomly but reproducibly using a fixed seed of 42, ensuring consistent comparisons across experiments. To enforce reproducibility, the same seed is applied across Python, NumPy, and PyTorch (central processing unit and Compute Unified Device Architecture), with deterministic CUDA deep neural network behavior enabled. During training, samples are loaded in batches of 32. The fusion backbone uses a shared projection dimension of 768, with 4 attention heads and 3 cross-attention layer pairs per fusion stage, and models are trained for up to 50 epochs with a dropout rate of 0.1. To reduce scale mismatch across modalities, we apply L2 normalization to the input embeddings prior to fusion.

The model parameters are optimized using AdamW with a learning rate of 0.0001 and weight decay of 1 × 10^−4^. For AD detection, we minimize cross-entropy loss, and for AD severity assessment, we use mean squared error loss. To stabilize learning, we use a ReduceLROnPlateau scheduler that monitors validation loss and reduces the learning rate by a factor of 0.5 after 2 consecutive epochs without improvement. We additionally apply gradient clipping with a maximum norm of 1.0. Early stopping is based on validation loss with a patience of 5 epochs, and the model weights from the epoch with the lowest validation loss are restored for final evaluation and embedding extraction.

### AD Detection Task

The AD detection task is formulated as a binary classification problem, where the goal is to distinguish between participants with AD and cognitively normal (non-AD) participants using picture-description responses from the ADReSSo dataset. Because dementia-related signals can manifest across multiple modalities, we evaluate models using (1) audio-only representations, (2) text-only representations, and (3) multimodal fusion representations derived from combinations of text, audio, and image. Audio representations are extracted from Wav2Vec 2.0 embeddings, text representations are extracted from ModernBERT embeddings computed over Whisper transcripts, and image representations are extracted using CLIP ViT-L/14. We primarily use 3 standard machine-learning models for the AD classification task. These include support vector classifier (SVC), random forest (RF), and logistic regression (LR), all of which are implemented from the scikit-learn package [22-25].

### AD Severity Assessment Task

In addition to AD classification, we evaluate our approach for AD severity assessment by predicting MMSE cognitive score, formulated as a regression problem. The objective is to predict a participant’s cognitive score directly from their picture-description response, enabling a finer-grained estimate of cognitive status beyond binary diagnosis. Similar to the classification setting, we investigate the predictive use of multiple modalities by training models with audio-only, text-only, and image-only representations, as well as multimodal fusion representations that combine complementary cues across modalities. Specifically, we use support vector regressor (SVR), random forest regressor (RFR), and ridge regression (Ridge) as our machine learning models [23-26]. For this task, we use the same feature representations described in the preprocessing pipeline: Wav2Vec 2.0 embeddings for audio, ModernBERT embeddings derived from Whisper transcripts for text, and CLIP ViT-L/14 embeddings for the image stimulus. We compare our proposed attention-based trimodal fusion model against unimodal baselines and bimodal fusion models.

### Evaluation Metrics

We evaluate performance separately for the AD classification and AD severity assessment tasks. For the binary classification setting, we report accuracy, precision, recall, and *F*_1_-score, providing a balanced view of overall correctness as well as performance on the positive AD class. Additionally, we also show the receiver operating characteristic (ROC) curve for modality comparisons and report the area under the ROC curve. For the regression task, we quantify prediction error using root mean squared error (RMSE), which measures the average magnitude of score prediction deviations. For all classical machine-learning baselines, optimal hyperparameters are selected using grid search with 5-fold cross-validation performed on the training split only. This ensures that model configuration is tuned in a statistically robust manner while keeping the held-out test set strictly reserved for final evaluation.

To better characterize uncertainty in model performance, we additionally report 95% CIs for the major classification and regression results using nonparametric bootstrap resampling of the held-out test set. For each metric, CIs were estimated from the empirical bootstrap distribution across 1000 repeated resamples. Note that the observed performance differences were not tested for statistical significance to avoid overreliance on arbitrary significance thresholds (eg, *P*<.05) and potential misinterpretations of *P* values.

### Attention Variant Study (Bidirectional Cross-Attention Versus Cross-Attention Only)

To assess the impact of reciprocal information exchange during multimodal fusion, we compare 2 attention configurations under an otherwise identical fusion framework. The cross-attention-only, namely standard cross-attention, performs fusion using a single cross-attention direction, where 1 modality attends to another to incorporate complementary information. In contrast, the bidirectional cross-attention applies cross-attention in both directions, allowing each modality to be updated using information from the other in a sequential manner. Both variants use the same pretrained embeddings, projection dimensionality, optimization settings, and train or validation split, enabling a controlled comparison of the attention mechanisms.

### Comparison with Conventional Fusion Methods

To assess whether the proposed attention-based multimodal fusion provides benefits beyond standard approaches, we compare it against 2 widely used conventional fusion strategies for the AD classification task: early fusion and late fusion. These baselines are commonly adopted in multimodal machine learning due to their simplicity, but they often struggle to capture complex interactions between modalities. For early fusion, we construct a single feature vector by concatenating the modality-specific embeddings (text, audio, and image) and train a classifier directly on the combined representation. While straightforward, early fusion does not explicitly model cross-modal relationships and instead relies on the downstream classifier to learn interactions implicitly from the concatenated embedding.

For late fusion, we train separate unimodal classifiers and combine their predictions at the decision level using majority voting. Because the visual stimulus is identical across all samples in the ADReSSo picture-description setting, an image-only classifier would not be meaningful for late fusion. Therefore, we report late-fusion performance using only the text and audio unimodal models. This comparison helps isolate the benefit of our attention-based fusion, which can jointly reason over modalities and learn cross-modal dependencies rather than combining independent unimodal decisions.

In addition to these conventional fusion strategies, we include a neural network baseline trained on the concatenated multimodal embeddings. The neural network architecture consisted of 2 hidden layers with 512 and 128 nodes, respectively, using the rectified linear unit activation function. This funnel-like bottleneck design by progressively stepping down from the high-dimensional input space to 512 and then 128 nodes was chosen to systematically compress the multimodal features. This encourages the network to learn robust, high-level representations while mitigating the risk of overfitting on the relatively small dataset [27]. The network was optimized using the Adam solver with an initial learning rate of 0.001. To further mitigate overfitting and ensure optimal generalization, we implemented early stopping. Specifically, 10% of the training data were set aside as a validation set, and training was terminated if the validation score did not improve by at least 1 × 10^−4^ for 10 consecutive epochs. This baseline provides a stronger learned comparison than linear or tree-based classifiers on early-fusion features, while still operating on the same underlying embedding inputs. In this way, we can assess whether any observed gains arise simply from using a more flexible classifier on concatenated features or from the proposed bidirectional fusion mechanism itself.

### Ethical Considerations

The ADReSSo Challenge data used in this study are available through DementiaBank with approved credentials. The studies involving human participants were reviewed and approved by the DementiaBank consortium. All enrolled participants provided informed written consent to participate in this study. All data analyses in this work were conducted using deidentified data.

## Results

### AD Detection Task

#### Unimodal Baselines

In Table 1, we compare the performance of text and audio embeddings using the different classifiers. From the table, we can see that text provides stronger prediction for AD than the audio. With ModernBERT embeddings, SVC attains an *F*_1_-score of 0.8334, outperforming LR which has an *F*_1_-score of 0.7910 and RF which has an *F*_1_-score of 0.7713. In general, audio (wav2vec2) is weaker across all the classifiers as the best audio-only result is achieved by RF with an *F*_1_-score of 0.6736 and an accuracy of 0.7048. This performance gap between text and audio underscores that picture-description transcripts capture more discriminative information than the audio embeddings.

**Table 1.** Unimodal baselines on the ADReSSo 2021 unseen test set (n=71)^a^.

Classifier	Accuracy (95% CI)	Precision (95% CI)	Recall (95% CI)	*F*_1_-score (95% CI)
SVC^b^_text_	0.8448 (0.7606‐0.9296)	0.8744 (0.7500‐0.9714)	0.7999 (0.6571‐0.9231)	0.8334 (0.7241‐0.9231)
SVC_audio_	0.7051 (0.5915‐0.8028)	0.7069 (0.5484‐0.8529)	0.6852 (0.5172‐0.8294)	0.6928 (0.5588‐0.8095)
LR^c^_text_	0.7876 (0.6901‐0.8732)	0.7618 (0.6190‐0.8919)	0.8275 (0.6969‐0.9429)	0.7910 (0.6774‐0.8889)
LR_audio_	0.6617 (0.5493‐0.7746)	0.6886 (0.5161‐0.8519)	0.5712 (0.3953‐0.7317)	0.6207 (0.4666‐0.7500)
RF^d^_text_	0.8029 (0.7042‐0.8873)	0.8886 (0.7500‐1.0000)	0.6859 (0.5294‐0.8333)	0.7713 (0.6429‐0.8772)
RF_audio_	0.7048 (0.5915‐0.8169)	0.7338 (0.5714‐0.8846)	0.6284 (0.4643‐0.7858)	0.6736 (0.5283‐0.7949)

a Performance of text-only (ModernBERT embeddings) and audio-only (wav2vec 2.0 embeddings) representations with 3 standard classifiers (support vector classifier, logistic regression, and random forest) is shown.

b SVC: support vector classifier.

c LR: logistic regression.

d RF: random forest.

#### Bimodal Fusion (2-Way)

Combining text and image embeddings yields consistently strong performance as shown in Table 2, reflecting the benefit of pairing transcript semantics with visual context from the Cookie Theft stimulus. Across classifiers, the results remain close to the text-only baselines, suggesting that the image modality provides incremental gain rather than a major boost in this setting. The top configuration is achieved by SVC, reaching an *F*_1_-score of 0.8371 with an accuracy of 0.8437, slightly exceeding the text-only SVC *F*_1_-score (0.8358). RF also performs competitively (*F*_1_-score=0.8428), while LR attains an *F*_1_-score of 0.8011. Overall, text continues to dominate prediction quality, while image representations appear to offer modest complementary signal.

**Table 2.** Text + image fusion for Alzheimer disease (AD) classification^a^.

Classifier	Accuracy (95% CI)	Precision (95% CI)	Recall (95% CI)	*F*_1_-score (95% CI)
SVC^b^	0.8437 (0.7465‐0.9296)	0.8509 (0.7241‐0.9655)	0.8275 (0.6944‐0.9429)	0.8371 (0.7333‐0.9247)
LR^c^	0.8011 (0.7042‐0.8873)	0.7978 (0.6571‐0.9231)	0.7987 (0.6571‐0.9259)	0.7959 (0.6857‐0.8919)
RF^d^	0.8428 (0.7606‐0.9296)	0.8731 (0.7500‐0.9714)	0.7987 (0.6571‐0.9286)	0.8321 (0.7241‐0.9231)

a Performance of bimodal text + image representations on the ADReSSo 2021 unseen test set (n=71) using 3 classifiers (support vector classifier, logistic regression, and random forest) is shown. Text embeddings are extracted with ModernBERT, and image embeddings are extracted with contrastive language–image pretraining ViT-L/14.

b SVC: support vector classifier.

c LR: logistic regression.

d RF: random forest.

The text + audio pairing produces the best bimodal performance in Table 3 among all 2-way combinations. In particular, incorporating audio improves performance beyond transcript-only models, suggesting that features such as speech rhythm, hesitation patterns, and fluency irregularities provide additional discriminative value. The best overall results are obtained with SVC, which achieves an accuracy of 0.8584 and an *F*_1_-score of 0.8503, outperforming both the text-only SVC model (*F*_1_-score=0.8358) and the other bimodal settings. RF remains strong (*F*_1_-score=0.8325), whereas LR performs slightly lower (*F*_1_-score=0.7903). These results support the benefit of integrating acoustic information when strong text representations are available.

**Table 3.** Text + audio fusion for Alzheimer disease (AD) classification^a^.

Classifier	Accuracy (95% CI)	Precision (95% CI)	Recall (95% CI)	*F*_1_-score (95% CI)
SVC^b^	0.8584 (0.7746‐0.9296)	0.8771 (0.7568‐0.9722)	0.8286 (0.6944‐0.9444)	0.8503 (0.7458‐0.9315)
LR^c^	0.8016 (0.7042‐0.8873)	0.8165 (0.6765‐0.9394)	0.7704 (0.6249‐0.9024)	0.7903 (0.6762‐0.8889)
RF^d^	0.8440 (0.7606‐0.9296)	0.8732 (0.7500‐0.9714)	0.7993 (0.6571‐0.9231)	0.8325 (0.7213‐0.9206)

a Performance of bimodal text + audio representations on the ADReSSo 2021 unseen test set (n=71) using 3 classifiers (support vector classifier, logistic regression, and random forest) is shown. Text embeddings are extracted with ModernBERT, and audio embeddings are extracted with Wav2Vec 2.0.

b SVC: support vector classifier.

c LR: logistic regression.

d RF: random forest.

Table 4 shows results for audio + image fusion, a pairing that excludes transcript information and relies only on acoustic patterns and visual context. Performance is notably lower than text-based bimodal models, but it improves substantially over audio-only baselines, suggesting that image features contribute useful grounding signals when linguistic embeddings are not available. The top performance is achieved by SVC, with an accuracy of 0.7188 and an *F*_1_-score of 0.6934, followed closely by RF (*F*_1_-score=0.6830). LR produces a slightly weaker result (*F*_1_-score=0.6530). Overall, these results reinforce that while audio + image alone can support AD prediction above chance, transcript-derived text embeddings remain critical for achieving top performance on picture-description based dementia screening.

**Table 4.** Audio + image fusion for Alzheimer disease (AD) classification^a^.

Classifier	Accuracy (95% CI)	Precision (95% CI)	Recall (95% CI)	*F*_1_-score (95% CI)
SVC^b^	0.7188 (0.6197‐0.8169)	0.7427 (0.5806‐0.8889)	0.6558 (0.4997‐0.8065)	0.6934 (0.5574‐0.8116)
LR^c^	0.6760 (0.5634‐0.7746)	0.6869 (0.5185‐0.8438)	0.6283 (0.4643‐0.7857)	0.6530 (0.5098‐0.7742)
RF^d^	0.7180 (0.6056‐0.8169)	0.7582 (0.6000‐0.9091)	0.6270 (0.4571‐0.7812)	0.6830 (0.5385‐0.8056)

a Performance of bimodal audio + image representations on the ADReSSo 2021 unseen test set (n=71) using 3 classifiers (support vector classifier, logistic regression, and random forest) is shown. Audio embeddings are extracted with Wav2Vec 2.0, and image embeddings are extracted with CLIP ViT-L/14.

b SVC: support vector classifier.

c LR: logistic regression.

d RF: random forest.

#### Multimodal Fusion (3-Way)

Using all 3 modalities (text, audio, and image) leads to the highest AD classification performance in our experiments. Among the 3 classifiers, SVC achieves the best overall results, with an accuracy of 0.8722 and an *F*_1_-score of 0.8667. LR attains an *F*_1_-score of 0.8542, while RF achieves an *F*_1_-score of 0.8371. Overall, the 3-way fusion setting produces strong and stable performance across classifiers, with SVC providing the highest classification scores (Table 5).

**Table 5.** Multimodal fusion for Alzheimer disease (AD) classification^a^.

Classifier	Accuracy (95% CI)	Precision (95% CI)	Recall (95% CI)	*F*_1_-score
SVC^b^	0.8722 (0.7887‐0.9437)	0.8806 (0.7632‐0.9730)	0.8564 (0.7317‐0.9688)	0.8667 (0.7692‐0.9474)
LR^c^	0.8582 (0.7746‐0.9437)	0.8558 (0.7250‐0.9677)	0.8563 (0.7273‐0.9678)	0.8542 (0.7536‐0.9367)
RF^d^	0.8437 (0.7465‐0.9296)	0.8509 (0.7241‐0.9655)	0.8275 (0.6944‐0.9429)	0.8371 (0.7333‐0.9247)

a Performance of text + audio + image fusion on the ADReSSo 2021 unseen test set (n=71) using 3 classifiers (support vector classifier, logistic regression, random forest). Text embeddings are extracted with ModernBERT, audio embeddings with Wav2Vec 2.0, and image embeddings with CLIP ViT-L/14.

b SVC: support vector classifier.

c LR: logistic regression.

d RF: random forest.

### ROC Curve Comparison Between Bimodal and Multimodal Fusion

**Figure 2.**
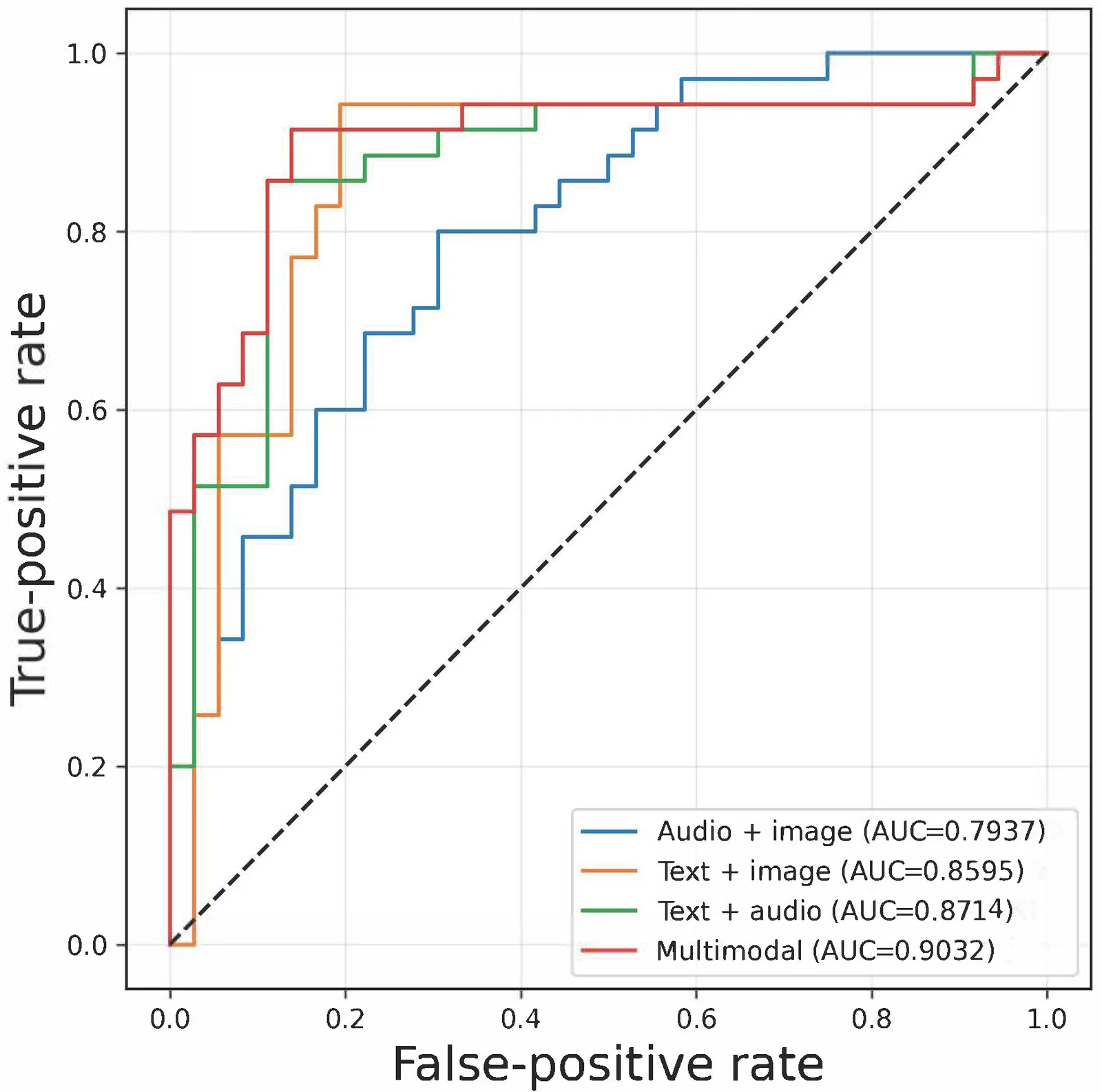
Receiver operating characteristic (ROC) comparison across bimodal and multimodal fusion (Alzheimer disease [AD] classification). ROC curves on the ADReSSo 2021 unseen test set (n=71) compare bimodal fusion models (audio + image, text + image, text + audio) against the proposed trimodal multimodal fusion (text + audio + image). The legend reports the area under the receiver operating characteristic curve (AUC) for each method.

Figure 2 compares ROC curves across bimodal fusion settings and the proposed trimodal multimodal fusion model for AD classification. Among the bimodal approaches, text + audio achieves the highest discrimination performance (area under the receiver operating characteristic curve [AUC]=0.8714), followed closely by text + image (AUC=0.8595), while audio + image performs notably lower (AUC=0.7937). The proposed multimodal fusion model yields the strong overall ROC performance with an AUC of 0.9032, indicating improved separability between participants with AD and without AD relative to all bimodal alternatives.

### Comparison to Other Fusion Methods

Table 6 compares the proposed multimodal fusion with conventional early fusion, late fusion, and a neural network baseline trained on concatenated embeddings. For SVC, both early fusion and late fusion achieve an accuracy of 0.8169, with *F*_1_-scores of 0.7937 and 0.8000, respectively. The neural network baseline improves this comparison to an accuracy of 0.8311 and an *F*_1_-score of 0.8099. In contrast, the proposed multimodal fusion achieves a higher accuracy of 0.8722 and a higher *F*_1_-score of 0.8667. A similar pattern is observed across the other classifier families, indicating that the advantage of the proposed fusion is not limited to comparison against only simple conventional baselines.

**Table 6.** Comparison with conventional and learned fusion baselines for Alzheimer disease (AD) classification^a^.

Fusion method	Accuracy	Precision	Recall	*F*_1_-score
SVC^b^_early fusion_	0.8169	0.8929	0.7143	0.7937
SVC_late fusion_	0.8169	0.8666	0.7428	0.8000
SVC_multimodal fusion_	0.8722	0.8806	0.8564	0.8667
LR^c^_early fusion_	0.8028	0.8387	0.7429	0.7429
LR_late fusion_	0.7887	0.8125	0.7428	0.7761
LR_multimodal fusion_	0.8582	0.8558	0.8563	0.8542
RF^d^_early fusion_	0.8028	0.8889	0.6857	0.7742
RF_late fusion_	0.7887	0.8333	0.7142	0.7692
Neural network	0.8311	0.8964	0.7427	0.8099
RF_multimodal fusion_	0.8437	0.8509	0.8275	0.8371

a Performance comparison between early fusion (feature concatenation), late fusion (decision-level fusion of text and audio), a neural network baseline trained on concatenated embeddings, and the proposed attention-based multimodal fusion (text + audio + image) on the ADReSSo 2021 unseen test set (n=71) is shown.

b SVC: support vector classifier.

c LR: logistic regression.

d RF: random forest.

### Bidirectional Cross-Attention Versus Cross-Attention Only

We evaluate 2 fusion variants to quantify the impact of the bidirectional cross attention. Across all 3 classifiers (Table 7), the bidirectional cross-attention configuration achieves stronger overall performance than cross-attention only. For SVC, cross-attention alone attains an *F*_1_-score of 0.8571 with accuracy of 0.8732, while our bidirectional cross attention improves results to an *F*_1_-score of 0.8667 and an accuracy of 0.8722. A similar trend is observed for LR. For RF, the cross-attention only baseline achieves an *F*_1_-score of 0.8125 (accuracy=0.8309), and the bidirectional cross-attention variant improves to an *F*_1_-score of 0.8371 (accuracy=0.8437). Overall, bidirectional cross-attention produces balanced AD classification performance metrics.

**Table 7.** Performance comparison between cross-attention–only fusion and the proposed bidirectional cross-attention fusion on the ADReSSo 2021 unseen test set (n=71) across 3 classifiers (support vector classifier [SVC], logistic regression [LR], random forest [RF]).

Fusion method	Accuracy	Precision	Recall	*F*_1_-score
SVC_cross_attention_	0.8732	0.9642	0.7714	0.8571
SVC_bidirectional_	0.8722	0.8806	0.8564	0.8667
LR_cross_attention_	0.8028	0.7837	0.8285	0.8055
LR_bidirectional_	0.8582	0.8558	0.8563	0.8542
RF_cross_attention_	0.8309	0.8965	0.7428	0.8125
RF_bidirectional_	0.8437	0.8509	0.8275	0.8371

### AD Severity Assessment Task

#### Overview

We evaluated how well individual modalities support continuous MMSE score prediction and compare them to pairwise and their multimodal counterparts (Table 8). Across all 3 modalities for regression, text embeddings again outperform audio. With ModernBERT features, Ridge regression attains the lowest text-only error, followed closely by SVR and RFR. In contrast, the corresponding audio-only models with wav2vec2 embeddings are substantially less accurate.

**Table 8.** Mini-Mental Status Examination (MMSE) score prediction across unimodal, bimodal, and trimodal fusion settings on the ADReSSo 2021 unseen test set (n=71)^a^.

Model and classifier	RMSE^b^ (95% CI)
Unimodal	
SVR^c^_text_	4.3959 (3.7870‐5.0445)
SVR_audio_	6.0146 (4.6954‐7.2511)
Ridge^d^_text_	4.5951 (3.7248‐5.4198)
Ridge_audio_	5.9914 (4.9110‐7.1118)
RFR^e^_text_	4.5806 (3.8630‐5.3138)
RFR_audio_	8.0297 (6.8131‐9.2356)
Pairwise	
SVR_text + image_	4.3897 (3.7793‐4.9916)
Ridge_text + image_	4.3333 (3.5186‐4.7430)
RFR_text + image_	4.4310 (3.6517‐5.2476)
SVR_audio + image_	6.0201 (4.6987‐7.2746)
Ridge_audio + image_	5.8611 (4.9170‐6.8695)
RFR_audio + image_	6.1440 (5.0956‐7.1654)
SVR_text + audio_	4.6745 (3.9837‐5.3725)
Ridge_text + audio_	4.3122 (3.7134‐4.9219)
RFR_text + audio_	4.7432 (4.0348‐5.4675)
Multimodal	
SVR_text + audio + image_	4.2134 (3.4898‐5.0544)
Ridge_text + audio + image_	4.1440 (3.5511‐4.7421)
RFR_text + audio + image_	4.3856 (3.8126‐5.0012)

a Root mean squared error with 95% CI is reported for unimodal models using text-only (ModernBERT embeddings) and audio-only (Wav2Vec 2.0 embeddings), bimodal fusion models using text + image, text + audio, and audio + image representations, and the proposed trimodal fusion model using text + audio + image. The results are shown for 3 regression heads: support vector regressor, ridge regression, and random forest regressor.

b RMSE: root mean squared error.

c SVR: support vector regressor.

d Ridge: ridge regression.

e RFR: random forest regressor.

For pairwise fusion, the lowest prediction error is achieved when text is included in the fusion pair, while combinations that exclude text remain substantially weaker. The best-performing bimodal configuration is Ridge with text + image, which attains the lowest RMSE of 4.3333, followed closely by SVR. These results indicate that integrating a second modality alongside text can reduce prediction error relative to text-only baselines. In contrast, pairings based on audio + image perform consistently worse across all regression heads.

Across the 3 regression heads, multimodal fusion achieves consistently low error, with Ridge obtaining the best overall RMSE of 4.1440, followed closely by SVR regression. RFR also performs competitively with an RMSE of 4.3856. Overall, the 3-way fusion setting yields strong and stable MMSE prediction accuracy across model families, with SVR and Ridge producing the lowest errors.

#### Comparison With Representative ADReSSo 2021 Multimodal and Transformer-Based Studies

To further position our method relative to recent benchmark-relevant approaches, we conducted a targeted scoping review of ADReSSo 2021 studies using multimodal, transformer, and attention-based methods. From this review, we selected e representative ADReSSo 2021 comparator studies for focused comparison: Wang et al [28], Zhu et al [29], Bang et al [30], and Shao and Fang [31]. These studies span attention-based multimodal fusion, wav2vec2 + Bidirectional Encoder Representations from Transformers (BERT) hybrid modeling, large language model–augmented multimodal modeling, and co-attention acoustic-text fusion, respectively. Table 9 summarizes their reported AD classification performance alongside our proposed model. Details of the scoping-review procedure are provided in Multimedia Appendix 1.

**Table 9.** Comparison with representative modern ADReSSo 2021 multimodal and transformer-based comparator models for Alzheimer disease (AD) classification^a^.

Study and year	Modalities	Model	Accuracy	*F*_1_-score
Wang et al [28], 2021	Linguistic + IS10 + X-Vector	DNN^b^	0.80	0.83
Zhu et al [29], 2021	Audio + ASR^c^ text	Wav2vec2 + BERT^d^	0.83	0.83
Bang et al [30], 2024	Audio + text + LLM^e^-generated “opinion” feature	ChatGPT-assisted multimodal model	0.87	0.87
Shao and Fang [31], 2025	Acoustic + ASR text	Co-attention multimodal model	0.83	0.84
Ours	Audio + text + image	Bidirectional attention + SVC	0.87	0.87

a The table reports a focused comparison between our proposed audio + text + image bidirectional attention framework and four representative ADReSSo 2021 studies selected from the targeted scoping review: Wang et al [28] (attention-based multimodal deep neural network), Zhu et al [29] (wav2vec2 + BERT), Bang et al [30] (large language model–augmented multimodal model), and Shao and Fang [31] (co-attention multimodal fusion). Accuracy and *F*_1_-score are reported as provided in the respective studies.

b DNN: deep neural network.

c ASR: automatic speech recognition.

d BERT: Bidirectional Encoder Representations from Transformers.

e LLM: large language model.

## Discussion

Our findings indicate that trimodal fusion of text, audio, and image provides the overall strong performance on ADReSSo for both AD classification and MMSE prediction. Relative to unimodal and bimodal alternatives, the trimodal setting yields the best overall classification performance and the lowest or near-lowest regression error across model families. Because ADReSSo was designed to reduce common confounds such as demographic imbalance, these gains are likely to reflect disease-relevant information rather than spurious correlations [8].

A consistent trend across the tasks is that text representations dominate unimodal performance, with transcript-derived embeddings outperforming audio-only models by a substantial margin. This aligns with longstanding clinical and computational evidence that connected speech contains measurable dementia-related changes in lexical selection, semantic content, and syntactic organization [32]. In our experiments, ModernBERT-based embeddings preserve much of this discriminative linguistic structure, yielding the strong unimodal baselines across both classification and MMSE prediction.

Although audio-only performance is weaker, audio contributes useful complementary information when paired with text, particularly for AD classification where text + audio is a rather competitive bimodal configuration and for MMSE prediction. This is consistent with prior work on paralinguistic and timing-related markers in dementia, such as pausing, hesitation, and fluency disruptions, which are not fully captured in transcripts alone [33]. The use of self-supervised acoustic representations such as wav2vec 2.0 provides a principled way to encode these cues from raw speech [19].

The image modality plays a more nuanced role in this benchmark. Because the Cookie Theft stimulus is fixed across participants, an image-only system is not expected to be discriminative; however, our results suggest that including image embeddings can still improve performance when used jointly with text and audio. One plausible explanation is that the image representation acts as a shared contextual anchor for the narrative. This interpretation is supported by several findings. First, drawing on the image-text matching approach [34], prior work [35] shows that healthy and dementia samples differ in their relevance to the target picture, with healthy samples yielding higher relevance scores. This finding suggests that visual information can contribute meaningfully to dementia detection. Second, we conducted a series of comparisons to evaluate the added value of the image modality (Tables 1–5), including text versus text + image, audio versus audio + image, and text + audio versus text + audio + image. Across all settings, incorporating the image leads to consistent, albeit modest, performance gains when combined with other modalities. Third, we performed a control analysis by randomly shuffling half of the image patches in the text + image fusion model. This manipulation resulted in a drop in performance relative to the original images (*F*_1_-score: 0.84 vs 0.79), providing direct evidence that preserving the visual structure is important for model effectiveness. Taken together, these results indicate that the image serves as a semantic anchor to leverage the full contextual information available. Since the same image is used for every participant, the study demonstrates the usefulness of shared stimulus grounding rather than the predictive value of participant-specific image data.

A key methodological advance from our ablation study is that bidirectional cross-attention consistently outperforms standard 1-way cross-attention across classifier families. This suggests that reciprocal information exchange between modalities is more effective than a single one-way update for this task. In other words, allowing each modality to attend to and be updated by the other leads to stronger fusion than unidirectional conditioning alone [12]. The comparison with early and late fusion further shows that how modalities are integrated matters. Early fusion and late fusion are simple and widely used, but they do not model cross-modal relationships as explicitly as attention-based intermediate fusion. Their weaker performance in our experiments supports the value of learned cross-modal interaction over heuristic fusion strategies [35].

Across tasks, the regression results mirror classification trends: text-only is the strongest among unimodal baselines, bimodal improvements are most pronounced when text is included, and trimodal fusion produces the lowest overall error. This consistency suggests that the multimodal embedding learned through attention mechanisms supports both discrete diagnostic labeling and continuous cognitive assessment, a useful property for clinical decision support where disease manifestations often exist on a spectrum rather than a binary boundary. At the same time, RMSE values remain nontrivial, highlighting that cognitive score prediction remains a challenging objective likely influenced by heterogeneity in impairment profiles and the limited size of available labeled data.

Beyond the in-benchmark comparisons reported in the *Results* section, Table 9 provides additional context against representative modern ADReSSo 2021 multimodal and transformer-based studies. As shown in Table 9, our method remains competitive relative to these comparator models. At the same time, direct one-to-one comparison remains imperfect even within the ADReSSo-only subset because studies differ in modality definitions, auxiliary cues, and evaluation protocols. We therefore use Table 9 as a focused benchmark-context comparison rather than as a strict ranked state-of-the-art table.

Several limitations should be considered when interpreting these findings. First, the findings have not yet been validated across an independent dataset, a different elicitation image, or a different clinical population. ADReSSo is a relatively small benchmark dataset for training and evaluating multimodal deep learning models, with 237 total samples and a held-out test set of 71 recordings. Although the dataset is carefully curated and demographically balanced, the limited sample size may constrain generalizability and increase the possibility that strong performance partly reflects benchmark-specific characteristics rather than broader robustness across populations or recording conditions. Accordingly, this study should be interpreted primarily as a benchmarked methodological investigation on ADReSSo that evaluates the potential of bidirectional multimodal fusion, rather than as definitive evidence of clinical generalization. Second, picture-description tasks represent a structured elicitation paradigm; performance may differ for more naturalistic conversational speech, where topic drift and dialogue dynamics introduce additional complexity. Third, transcript-based modeling depends on automatic speech recognition quality; while Whisper-style transcription is robust in many conditions, transcription choices around disfluencies and fillers can influence downstream embedding behavior especially in tasks where such markers may carry clinical signal.

Finally, these results suggest several directions for future work. More fine-grained alignment strategies (eg, segment-level coupling between audio and text representations) may strengthen the ability of cross-attention to learn clinically meaningful correspondences. Additionally, evaluation across multiple elicitation tasks and datasets would help clarify whether the observed gains extend beyond Cookie Theft.

In this work, we introduced an attention-based trimodal fusion framework for dementia screening that integrates text, audio, and image through bidirectional attention. Evaluated on the ADReSSo Cookie Theft picture-description benchmark, the proposed approach achieved overall strong performance for both AD classification and MMSE prediction, outperforming unimodal baselines, bimodal fusion models, and conventional early- and late-fusion strategies. The ablation analysis further showed that bidirectional cross-attention performs better than standard 1-way cross-attention, highlighting the value of reciprocal cross-modal interaction for dementia-related prediction.

## Supplementary material

10.2196/93279 Multimedia Appendix 1 Details of the scoping review procedure.

## Abbreviations

AD: Alzheimer disease
AUC: area under the receiver operating characteristic curve
BERT: Bidirectional Encoder Representations from Transformers
CLIP: contrastive language–image pretraining
LR: logistic regression
MMSE: Mini-Mental Status Examination
RF: random forest
RFR: random forest regressor
Ridge: ridge regression
RMSE: root mean squared error
ROC: receiver operating characteristic
SVC: support vector classifier
SVR: support vector regressor
