# Supplementary material for: Multimodal Dementia Prediction With Large Language Models: Cross-Attention Over Text, Audio, and Image
Source: JMIR Aging. 2026 Sep 21;9:e93279. doi: 10.2196/93279 (PMC13592564; doi:10.2196/93279)
Supplement: Supplementary file 1 [file aging-v9-e93279-s001.docx]

Targeted Review of ADReSSo 2021 Multimodal and Transformer-Based Studies

Felix Agbavor^1^, Hualou Liang^1,2,3,4†^

^1^	School of Biomedical Engineering, Science and Health Systems, Drexel University

^2^	Division of Artificial Intelligence and the Humanities, The Hong Kong Polytechnic University

^3^	Department of Language Science and Technology, The Hong Kong Polytechnic University

^4^	Departments of Data Science and Artificial Intelligence, The Hong Kong Polytechnic University

^†^	Correspondence: hualou.liang@polyu.edu.hk

We conducted a targeted scoping review centered on the ADReSSo 2021 [1] benchmark and its citation network in order to better position the present study relative to recent multimodal and transformer-based work. As of April 23, 2026, Google Scholar listed 287 citing records for the ADReSSo benchmark paper. Starting from this broad citation pool, we applied benchmark-centered keyword screening using terms such as multimodal, cross-attention, transformer, fusion, and Cookie Theft picture to identify studies most relevant to our setting. This initial screening narrowed the literature to 90 benchmark-adjacent candidate papers. This initial screening narrowed the literature to 90 benchmark-adjacent candidate papers. We then performed a second-stage screening to retain only studies that explicitly used the ADReSSo 2021 dataset, rather than papers that merely cited the benchmark or used related datasets such as ADReSS 2020. On this basis, we retained 18 ADReSSo-using papers with sufficient evidence for structured inclusion in this review. This process was designed to identify benchmark-relevant modern comparator models rather than to provide an exhaustive review of all dementia-detection studies.

The retained ADReSSo 2021 literature shows that directly relevant comparator studies do exist, and that the benchmark space had already begun moving beyond purely classical machine-learning pipelines as early as 2021. Among the earlier ADReSSo papers, Pan et al. [2] used ASR-confidence-guided BERT together with acoustic modeling, Pappagari et al. [3] combined x-vectors and prosody with BERT score fusion, and Rohanian et al. [4] proposed gated multimodal BiLSTM-style fusion over acoustic, lexical, pause, and disfluency cues. Qiao et al. [5] combined pretrained language models with disfluency-oriented text features, while Gauder et al. [6] provided a pretrained speech-embedding audio baseline, Chen et al.[7] systematically compared acoustic, linguistic, and fusion-based approaches on ADReSSo. Wang et al. [8] introduced an attention-based multimodal network over audio and language data, Syed et al.[9] proposed silence-aware multimodal fusion, and Zhu et al.[10] developed WavBERT, a wav2vec2+BERT hybrid. Considered together, these studies show that the ADReSSo comparison landscape already included attention-based, pretrained, and multimodal architectures, rather than being limited only to shallow classifiers or handcrafted-feature pipelines.

More recent ADReSSo-using work extends this benchmark landscape toward contemporary transformer and multimodal architectures. Ilias et al.[11] introduced context-aware attention layers coupled with optimal-transport domain adaptation and multimodal fusion across ADReSS and ADReSSo, while Yang et al.[12] reported a self-attention and cross-modal attention framework that included ADReSSo results. Bang et al.[13] incorporated an LLM-generated ‘opinion’ feature alongside audio and text in a ChatGPT-assisted multimodal model, Casu et al.[14] combined a fine-tuned LLM with acoustic features, and Shao and Fang[15] proposed a co-attention multimodal model over acoustic and ASR-transcribed text. Li and Zhang[16] further extended this benchmark line with Whisper-based transfer learning using full transcripts as prompts, showing that newer ASR- and transformer-driven approaches have also entered the ADReSSo comparison space. Pérez-Toro et al. [17] are also relevant in this context because they examined interviewer-related confounds within the ADReSSo benchmark, underscoring that stronger model classes must still be interpreted carefully when benchmark artifacts may exist. In parallel, Azadmaleki et al.[18] used ADReSSo as external validation within the SpeechCARE framework, and Liao et al.[19] used ADReSSo as benchmark-side validation within a broader longitudinal dual-modal framework. Taken together, these studies show that the recent ADReSSo literature now includes attention-based, transformer-oriented, and LLM-augmented multimodal systems rather than only early fusion, late fusion, or shallow classifier pipelines.

At the same time, not all ADReSSo-using studies are equally suitable for one-to-one comparison in the main manuscript. Some papers, such as Pérez-Toro et al.[17], are methodologically important but focus primarily on confound analysis rather than on proposing a standard benchmark model. Others, such as Ilias et al. [11] and Yang et al.[12], include ADReSSo results but do so in mixed-dataset settings, which makes them less clean as standalone ADReSSo comparisons. Azadmaleki et al.[18] and Liao et al.[19] also use ADReSSo in more indirect ways, as external or benchmark-side validation within broader multilingual or longitudinal frameworks rather than as the primary standalone AD classification benchmark. These differences matter because improved results can reflect a different modality budget, a different evaluation protocol, or a different experimental objective rather than a strictly better ADReSSo diagnosis model. For this reason, we did not treat all retained ADReSSo-using papers as equally comparable in the main manuscript.

From this retained set, we selected four representative papers for direct comparison in the main manuscript: Wang et al. (2021), Zhu et al. (2021), Bang et al. (2024), and Shao and Fang (2025). These studies were chosen because they all explicitly use ADReSSo 2021 and together span the main modern comparator categories most relevant to our work: an attention-based multimodal network (Wang et al.), a transformer-style audio-text hybrid based on wav2vec2 and BERT (Zhu et al.), an LLM-augmented multimodal system (Bang et al.), and a co-attention acoustic-text fusion model (Shao and Fang). Table 9 summarizes this focused comparison and is intended to contextualize our results against modern benchmark-relevant methods rather than to serve as a strict ranked state-of-the-art table.

Even after restricting the review to papers that actually use ADReSSo 2021, the retained literature remains substantially heterogeneous. The first source of heterogeneity is modality budget. Some papers use audio only, some use audio + ASR text, some incorporate image + text, and the newest systems use image + text + audio or even audio + text + LLM-generated feedback features. This means “multimodal ADReSSo” does not refer to one single experimental setting. The second source is evaluation protocol. The cleanest benchmark-aligned studies report one result on the official unseen ADReSSo test set. Other retained studies use 5-fold CV on the training data with majority-vote inference on the test set, mixed ADReSS / ADReSSo experiments, or external validation on ADReSSo after training elsewhere. Those papers do use ADReSSo, but they are not equally comparable. The third source is task framing. Some papers target binary AD classification, others jointly report MMSE regression or cognitive-decline prediction, and a few later papers use ADReSSo only to validate a framework whose main scientific aim is multilingual screening, MCI detection, or confound analysis. That variation matters because improved results can reflect a different protocol, a different subtask emphasis, or a different feature budget rather than a strictly better ADReSSo diagnosis model.

Taken together, this supports a balanced reviewer-facing conclusion: there is a real body of directly relevant multimodal and transformer work on ADReSSo 2021, but it is smaller than the full citation network and remains heterogeneous enough that broad ranking claims should be made cautiously.

**References**

1.	Luz S, Haider F, de la Fuente S, Fromm D, MacWhinney B. Detecting cognitive decline using speech only: The ADReSSo Challenge. *arXiv preprint arXiv:210409356*. Published online 2021.

2.	Pan Y, Mirheidari B, Harris J, et al. *Using the Outputs of Different Automatic Speech Recognition Paradigms for Acoustic- and BERT-Based Alzheimer’s Dementia Detection Through Spontaneous Speech*. 2021:3814. doi:10.21437/Interspeech.2021-1519

3.	Pappagari R, Cho J, Joshi S, et al. *Automatic Detection and Assessment of Alzheimer Disease Using Speech and Language Technologies in Low-Resource Scenarios*. 2021:3829. doi:10.21437/Interspeech.2021-1850

4.	Rohanian M, Hough J, Purver M. Alzheimer’s Dementia Recognition Using Acoustic, Lexical, Disfluency and Speech Pause Features Robust to Noisy Inputs. *arXiv*. Preprint posted online June 29, 2021:arXiv:2106.15684. Accessed July 11, 2023. http://arxiv.org/abs/2106.15684

5.	Qiao Y, Yin X, Wiechmann D, Kerz E. Alzheimer’s Disease Detection from Spontaneous Speech Through Combining Linguistic Complexity and (Dis)Fluency Features with Pretrained Language Models. In: *Interspeech 2021*. ISCA; 2021:3805-3809. doi:10.21437/Interspeech.2021-1415

6.	Gauder L, Pepino L, Ferrer L, Riera P. Alzheimer Disease Recognition Using Speech-Based Embeddings From Pre-Trained Models. In: *Interspeech 2021*. ISCA; 2021:3795-3799. doi:10.21437/Interspeech.2021-753

7.	Chen J, Ye J, Tang F, Zhou J. Automatic Detection of Alzheimer’s Disease Using Spontaneous Speech Only. *Interspeech*. 2021;2021:3830-3834. doi:10.21437/interspeech.2021-2002

8.	Wang N, Cao Y, Hao S, Shao Z, Subbalakshmi KP. Modular Multi-Modal Attention Network for Alzheimer’s Disease Detection Using Patient Audio and Language Data. In: *Interspeech 2021*. ISCA; 2021:3835-3839. doi:10.21437/Interspeech.2021-2024

9.	Syed ZS, Syed MSS, Lech M, Pirogova E. Tackling the ADRESSO Challenge 2021: The MUET-RMIT System for Alzheimer’s Dementia Recognition from Spontaneous Speech. In: *Interspeech 2021*. ISCA; 2021:3815-3819. doi:10.21437/Interspeech.2021-1572

10.	Zhu Y, Obyat A, Liang X, Batsis JA, Roth RM. WavBERT: Exploiting Semantic and Non-Semantic Speech Using Wav2vec and BERT for Dementia Detection. In: *Interspeech 2021*. ISCA; 2021:3790-3794. doi:10.21437/Interspeech.2021-332

11.	Ilias L, Askounis D. Context-aware attention layers coupled with optimal transport domain adaptation and multimodal fusion methods for recognizing dementia from spontaneous speech. *Knowledge-Based Systems*. 2023;277:110834. doi:10.1016/j.knosys.2023.110834

12.	Yang X, Hong K, Zhang D, Wang K. Early diagnosis of Alzheimer’s Disease based on multi-attention mechanism. *PLOS ONE*. 2024;19(9):e0310966. doi:10.1371/journal.pone.0310966

13.	Bang JU, Han SH, Kang BO. Alzheimer’s disease recognition from spontaneous speech using large language models. *ETRI Journal*. 2024;46(1):96-105. doi:10.4218/etrij.2023-0356

14.	Casu F, Lagorio A, Ruiu P, Trunfio GA, Grosso E. Integrating Fine-Tuned LLM with Acoustic Features for Enhanced Detection of Alzheimer’s Disease. *IEEE J Biomed Health Inform*. 2025;PP. doi:10.1109/JBHI.2025.3566615

15.	Shao Y, Fang T. Alzheimer’s Disease Detection Using Co-Attention Mechanism for Acoustic and ASR-Transcribed Text Features. In: *Interspeech 2025*. ISCA; 2025:5673-5677. doi:10.21437/Interspeech.2025-219

16.	Li J, Zhang WQ. Whisper-Based Transfer Learning for Alzheimer Disease Classification: Leveraging Speech Segments with Full Transcripts as Prompts. In: *ICASSP 2024 - 2024 IEEE International Conference on Acoustics, Speech and Signal Processing (ICASSP)*. 2024:11211-11215. doi:10.1109/ICASSP48485.2024.10448004

17.	Pérez-Toro PA, Bayerl SP, Arias-Vergara T, et al. Influence of the Interviewer on the Automatic Assessment of Alzheimer’s Disease in the Context of the ADReSSo Challenge. In: *Interspeech 2021*. ISCA; 2021:3785-3789. doi:10.21437/Interspeech.2021-1589

18.	Azadmaleki H, Haghbin Y, Rashidi S, Momeni Nezhad MJ, Zolnour A, Zolnoori M. SpeechCARE: dynamic multimodal modeling for cognitive screening in diverse linguistic and speech task contexts. *npj Digit Med*. 2025;8(1):677. doi:10.1038/s41746-025-02026-x

19.	Liao YS, Wai T, Liao TY, Chang HL, Chang YL, Fu LC. Mild Cognitive Impairment Detection System Based on Unstructured Spontaneous Speech: Longitudinal Dual-Modal Framework. *JMIR Med Inform*. 2026;14:e80883. doi:10.2196/80883
